# Intraventricular ejection pressure gradient derived from acceleration measurement by phase contrast CMR as a new marker of left ventricular contractility: feasibility study and preliminary results in dilated cardiomyopathy

**DOI:** 10.1186/1532-429X-15-S1-E6

**Published:** 2013-01-30

**Authors:** Laurent Macron, Emilie Bollache, Nadjia Kachenoura, Alain De Cesare, Golmehr Ashrafpoor, Arshid Azarine, Alban Redheuil, Elie Mousseaux

**Affiliations:** 1 Radiology, Cardiovascular Imaging Unit, HEGP, APHP, Paris, France; 2 LIF INSERM U678, UPMC, Paris, France

## Background

Intra-ventricular pressure gradient along the direction of the outflow tract is generated by the impulse response of the myocardial contraction. This spatial pressure gradient, derived from the maximal acceleration estimates within the left ventricular (LV) ejection flow, has been validated in vitro by using MRI and was found strongly related to the inotropic state of the LV as demonstrated in animal and human echocardiographic studies. This acceleration may be less dependant on load conditions than others LV systolic function parameters, especially LV ejection fraction (LVEF).

Aim: (1) to evaluate the feasibility of LV ejection flow acceleration (Acc) estimates by spatial and temporal derivative of velocities recorded by CMR using phase contrast sequence, (2) to compare the acceleration values to LVEF in patients with Dilated Cardiomyopathy (LVEF<50%) and in control subjects.

## Methods

Ten DCM patients (54±12 years, 60%men) and 14 age-matched controls underwent CMR with cine sequences to determine LV volumes and LVEF. Through plane phase contrast acquisitions were obtained in breathhold (TR = 7.5 ms, TE = 2.5 ms, 2 views per segment, temporal resolution = 15 ms, FOV 32 to 36 cm with 126 x 256 matrix) at 2 consecutive levels, at the level of aortic valve and 10 mm below through the LV outflow tract. Total Acceleration (TotalAcc) and its two components, Temporal (TempAcc) and Convective (ConvAcc) Acceleration were derived from velocities at phase contrast sequence by using our home-made previously validated semi-automatic software Art Fun.

## Results

Acceleration measurement was feasible in all DCM and controls. Overall, TempAcc (r=0.50, p=0.01) but not ConvAcc (r=0.33, p=0.11) correlated with LVEF. Nevetheless, TotalAcc was better correlated to LVEF (r=0.71, p=0.0001) than TempAcc derived from velocities obtained at the level of the aortic valve alone. Compared to control, mean LVEF (30±13 vs. 66±6, p<0.0001) TotalAcc (880±463 vs 1909±637 cm.s^-2^, p<0.001) and TempAcc (660±399 vs 1294±574 cm.s^-2^, p<0.01) but not ConvAcc (706±508 vs 1044±747 cm.s^-2^, p=0.22) were significantly lower in DCM.

## Conclusions

This preliminary study demonstrated the feasibility of LV ejection flow acceleration measurement derived from velocities at phase contrast sequence by CMR. TotalAcc is highly correlated to LVEF, better than TempAcc alone underlying the incremental value of ConvAcc.

## Funding

none

